# Characterising Free-Range Layer Flocks Using Unsupervised Cluster Analysis

**DOI:** 10.3390/ani10050855

**Published:** 2020-05-15

**Authors:** Terence Zimazile Sibanda, Mitchell Welch, Derek Schneider, Manisha Kolakshyapati, Isabelle Ruhnke

**Affiliations:** 1 School of Environmental and Rural Science, Faculty of Science, Agriculture, Business and Law, University of New England, Armidale, NSW 2351, Australia; mkolaksh@myune.edu.au (M.K.); iruhnke@une.edu.au (I.R.); 2 Precision Agriculture Research Group, Faculty of Science, Agriculture, Business and Law, School of Science and Technology, University of New England, Armidale, NSW 2351, Australia; mwelch8@une.edu.au (M.W.); dschnei5@une.edu.au (D.S.)

**Keywords:** aviary, eggs, individual, pasture, poultry, radio frequency identification (RFID), variation, spatial, technology, time budget, welfare

## Abstract

**Simple Summary:**

Little is known on how free-range laying hens on commercial farms exploit their offered resources. However, only when hen usage of the structural resources is understood, can design improvements be made to optimize hen health and welfare. This study was conducted in order to understand the extent to which free-range hens use the aviary system and range. With the help of individual tracking technology, agglomerative, and K-means cluster analysis, we were able to characterize various flock sub-populations. Regardless of the cluster group, hens used the nest boxes and lower feeder tier more consistently compared to the outdoor range and the upper feeder tier. Overall, hens that were more consistent with their average time spent at each location stayed for longer duration at each location than those hens that had inconsistent movement patterns. The identification of ‘routine’ behavior patterns can be essential for flock management, such as smothering prevention and future shed design.

**Abstract:**

This study aimed to identify sub-populations of free-range laying hens and describe the pattern of their resource usage, which can affect hen performance and welfare. In three commercial flocks, 3125 Lohmann Brown hens were equipped with radio-frequency identification (RFID) transponder leg bands and placed with their flock companions, resulting in a total of 40,000 hens/flock. Hens were monitored for their use of the aviary system, including feeder lines, nest boxes, and the outdoor range. K-means and agglomerative cluster analysis, optimized with the Calinski-Harabasz Criterion, was performed and identified three clusters. Individual variation in time duration was observed in all the clusters with the highest individual differences observed on the upper feeder (140 ± 1.02%) and the range (176 ± 1.03%). Hens of cluster 1 spent the least amount time on the range and the most time on the feed chain located at the upper aviary tier (*p <* 0.05). We conclude that an uneven load on the resources, as well as consistent and inconsistent movement patterns, occur in the hen house. Further analysis of the data sets using classification models based on support vector machines, artificial neural networks, and decision trees are warranted to investigate the contribution of these and other parameters on hen performance.

## 1. Introduction

Automated monitoring of animal behavior and welfare using sensor technology can offer valuable information in observing feeding, nest box usage, and ranging behavior. This is especially relevant for non-cage housing systems, where the opportunity for hens to express a wide range of behaviors can result in welfare concerns, such as severe feather pecking, cannibalism, smothering, and/or major injuries resulting in death [1]. The use of sensor technology, such as radio frequency identification (RFID), accelerometers, optic flow patterns, or thermographic cameras, has offered objective solutions to understand how birds use available infrastructure through the capability to collect hen movement data with time stamps for every second of the day, for every day of a hen’s life [2,3,4,5,6]. The increasing availability of these affordable tracking technologies has made it possible to collect very large, multi-dimensional datasets that provide a rich insight into the behavior of hens in the production system and their responses to physiological process that underpin their growth and development. These larger, richer datasets (and the potential for these to be collected in real-time) allow for the application of more sophisticated forms of analysis, such as the use of machine learning models for prediction and classification tasks. The overarching aim of introducing the machine learning approach to task monitoring the behavior/movement at the individual level is to provide producers with the potential for real-time flock monitoring and decision support that can be extended for risk evaluation, event prediction, and disease sensing [7].

The practicability of numerous technologic solutions to be adapted for poultry has been shown in research facilities but also under semi-commercial conditions [3,4,5,6,7,8]. By matching individual hen movement and body weight with nest box access, RFID systems have also demonstrated their value in recording individual hen performance [5,9,10].

Cluster analysis is an example of an unsupervised machine learning approach that is designed to assign random data into groups (clusters) to identify common patterns and improve understanding [11]. A cluster can be defined as a group of animals positioned or occurring closely together, also expressing similar behaviors at various times. More specifically, high dimensional cluster analysis can identify homogenous group behaviors not previously known because it is exploratory, which means it does not differentiate between dependent and independent variables.

High dimensional clustering can, therefore, be used as a reliable tool to investigate the usage and competition of resources, with direct implication to productivity and welfare of the hens. For example, smothering, frequently occurring at nest boxes or other structures, has been observed to cause up to 40% of the total flock mortality and approximately 10% overall mortality, resulting of an economic loss of up to 500,000 AUD/year (including loss of eggs) for an average egg producer in Australia with an average of 350,000 hens on-farm [12,13]. Commercial layers tend to cluster around key resources (feed, nest boxes) but also group according to their range use [4,14]. Hens can be described as low, medium, and high range users, according to the time they spend on the range or the number of days they visited the range. This time spent on the range is, however, correlated to the time that these hens spent at various areas within the shed [6]. Little information about potential sub-populations regarding in-shed resources or hens that exhibit specific movement patterns have been reported to-date. It is imperative to understand the social dynamics of free-range hens as it has a direct implication on the welfare of hens. Grouping of hens based on movement might be caused by social cohesion around using resources, such as feeders, nest boxes, and the range, or simply due to the presence and leadership of “alpha-hens” which are yet to be identified [14,15,16,17,18]. Insight in the dynamics and movement patterns of non-caged layers will give flock managers the ability to develop strategies to intervene with unfavorable behavior, prevent adverse events, and support positive choices that the hens are making. With near-real-time data processing, as well as temporal change detection, intervention could be performed based on an alert system. To further understand how hens use the aviary system and the outdoor provided to them, a fundamental query is whether different sub-populations of hens have different production performances, energy needs, behavioral repertoires, recognize resources in a different way, or have spatial abilities to use resources effectively.

Therefore, the aims of this study were (1) to identify hen clusters (based on movement data) in commercial free-range flocks provided with horizontal and vertical space, and (2) to investigate the variation of the movement patterns (duration) of the clusters regarding the various specific resources (aviary feed chains, nest boxes and the range) throughout the different production periods.

## 2. Materials and Methods

### 2.1. Ethical Statement

All procedures carried out in this study were approved by the University of New England’s Animal Ethics Committee (AEC 16-087).

### 2.2. Study Population and RFID Monitoring of Aviary and Range Usage 

**Figure 1:**
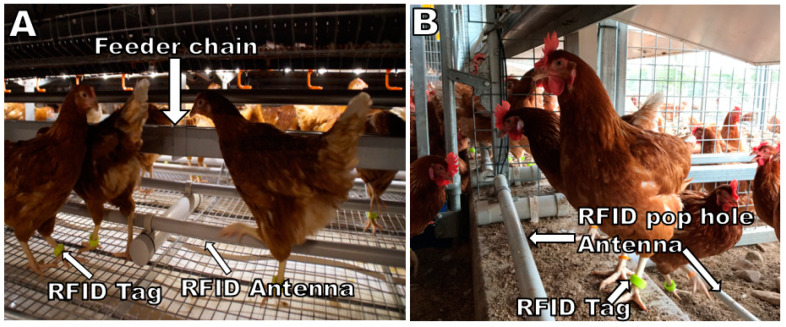
A picture showing the hens at the lower feeder tier (**A**) and the pop hole antenna (**B**). RFID = Radio-Frequency Identification.

Hen movement of three commercial free-range layer flocks was investigated. In each of the three flocks, 3125 Lohmann Brown hens aging 16 weeks were randomly selected, equipped with a transponder leg band and placed with their flock companions in five pens, resulting in a total of 625 hens/pen. The leg bands contained a RFID transponder (Monza R6 UHF RFID Tags, Impinj, Seattle, WA, USA) and a visual identification number printed on the outside. In each of three sheds, the three-tier aviary systems (two systems running parallel next to each other across the lengths of the shed) were modified with custom-made RFID antennae, which allowed monitoring of the tagged hens [6]. All hens were obtained from the same hatchery, placed at the same age, and managed by the same personnel in identical designed and equipped sheds. In the layer house, the 3125 tagged hens were placed in a subdivided area, where lateral cross-sectional partitioning of the equipment allowed equal access to all features of the shed, while at the same time being restricted to access the entire shed. This was done to allow for RFID instalment and intensive monitoring of the 3125 research hens, representing the movement of the entire flock. The restricted area was also extended to the range, where hens could access the range in the same manner as their flock companions but could not leave their enclosures to mix with un-tagged hens and enter an unmonitored area. The custom-built RFID antennae were placed along the entire length of the pop holes (total monitored length = 18 m), along the entire length of the partitioned range section (18 m), along the entire length of all nest box entries (18 m for each row of nest boxes), and along the entire length of the feeder chains (18 m along each side of the feeder chain). While the nest boxes were located in the middle tiers of the three-tier aviary system, they could only be entered from one side. In contrast, feeder chains were located at the bottom and top tiers and could be accessed from either the pop hole-facing side, as well as the shed-centre facing side. Therefore, two antennae were placed within 15 cm distance to each other along the right and left side of the feeder chain, which allowed the detection of hens that accessed the feeder chain regardless from which side they approached the feed (Figure 1). Further details can be found in Sibanda et al. (2019) [6]. The tagged hens in the monitored area experienced the same stocking density (indoor: 9 hens/m^2^; outdoor 1500 hens/ha) and the same management resources (lighting, nest box access, feed, feeding times, drinker set up, medications, etc.) as their flock companions. Hen movement data was collected after the hens were acclimatized to their new environment, when being 18 weeks of age, until 22 weeks of age.

### 2.3. Primary Data Collection

The data used in the experiment were collected from three separate flocks during December 2016 until June 2018. While, initially, 9375 individual hens were placed and monitored, only data obtained from 7244 hens were included in this research as these hens were still available at 74 weeks of age, when we obtained final body weight and egg follicle scores. The loss of experimental animals was due to RFID tag loss, RFID tag malfunction, and hen mortality. For the data analysis, we used eight variables for each hen, consisting of mean duration time at the lower feeder chain and the upper feeder chain, the nest box, the range (four variables), body weight at 16, 22, and 74 weeks of age (three variables), and egg follicle score at 74 weeks of age. The data were tested for normality using the Shapiro-Wilk test in JMP, version 14, SAS Institute Inc., Cary, NC, USA, 1989–2019, and non-parametric statistics were used to analyze the data.

### 2.4. Cluster Optimisation

Before revealing hidden clusters of similar mean daily duration in the different areas, the optimum number of clusters had to be established. In this study, the Calinski-Harabsz criterion was used to determine the optimum number of clusters without external information [19]. The Calinski-Harabsz is a variance ratio criterion that is maximized to provide an optimal clustering solution that takes into account the variance for data points within a cluster and the distance between clusters. The mean time each hen spent at each of the four different areas was used as the input for the calculation of the Calinski-Harabasz criterion. The Calinski-Harabasz criterion is defined in Equation (1) [19,20], as follows:

(1) $${\mathrm{CH}}_{k}\;=\frac{\mathrm{BGSS}}{k-1}\;÷\;\frac{\mathrm{WGSS}}{\left(n\;-\;k\right)},$$

where BGSS is represented by Equation (2), the overall between-cluster variance,

(2) $$\mathrm{BGSS}\;=\sum_{i=1}^{k}\;n_{i}{\left||m_{i}-m|\right|}^{2},$$

and WGSS represented by Equation (3), the overall within-cluster variance,

(3) $$\mathrm{WGSS}\;=\sum_{i=1}^{k}\sum_{x}^{k}{\left||m_{i}-m|\right|}^{2},$$

where:

CH = Calinski-Harabsz critetion

BGSS = between-group dispersion

WGSS = the pooled within-cluster sum of squares 

k = the number of clusters;

N = the number of observations;

*n_i_* = number of observations in cluster i;

*m_i_* = the centroid of cluster i;

*m* = overall mean of sample data;

||*m_i_ − m*|| = L^2^ norm (Euclidean distance);

*x* = data point;

*c_i_* = cluster i; and

||*x − m_i_*|| = L^2^ norm (Euclidean distance) between the two vectors.

### 2.5. Identifying Subpopulations Using K-Means and Agglomerative Clustering

Although the hens were monitored daily for 24 h, the calculation of the total daily access of the different areas included only the time from 4 am when the lights were switched on to 8 pm when the lights were switched off. The mean time that each hen spent at each one of the different zones was calculated as:

(4) $$\bar{x_{t}}=\frac{\mathrm{total}\;\mathrm{mins}\;\mathrm{spent}\;\mathrm{in}\;\mathrm{each}\;\mathrm{zone}\;}{\mathrm{total}\;\mathrm{number}\;\mathrm{of}\;\mathrm{days}\;\mathrm{an}\;\mathrm{individual}\;\mathrm{hen}\;\mathrm{accessed}\;\mathrm{each}\;\mathrm{area}},$$

where:

$\bar{x_{t}}$ = cluster mean;

t = time

The mean time the hens spent in each zone per day was used for the clustering analysis, resulting in a four-dimensional dataset. To validate the sub-populations composition, two clustering algorithms, the K-means and agglomerative hierarchical cluster analysis, were performed based on the mean daily duration data. The K-means algorithm is a well-established and widely used clustering approach that uses iterative refinement to partition the dataset into *k* clusters such that the sum of the squared distance between the data points of a cluster and its centroid is minimized [11].

K-means clustering uses a random initial state and will necessarily find the optimal solution. *k* (the number of clusters) is selected by maximizing the Calinski-Harabsz criterion. The agglomerative hierarchical clustering algorithm builds a hierarchy of clusters by starting with each data point as its cluster and successively merging points (according to a linkage criteria) until the desired number of clusters, *k*, is reached [21]. As with the K-means approach, the optimal solution is also selected by maximizing the Calinski-Harabsz criterion. The optimal clustering solutions were generated using built-in routines in MATLAB and Statistics Toolbox Release 2019a, (The MathWorks, Inc., Natick, MA, USA). After clustering the hens, *k* agreement statistics and a contingency table were used to evaluate the agreement between the two chosen methods by comparing individual hen clustering solution using the agglomerative and K-means clustering algorithms (JMP statistical software, version 14, SAS Institute Inc., Cary, NC, USA, 1989–2020).

### 2.6. Visualisation of the Clusters

To understand the data, the clustering solutions produced were analyzed using the dimensionality reduction algorithm *t*-distributed Stochastic Neighbor Embedding (*t*-SNE). *t*-SNE is a technique for visualizing high-dimensional data using a non-linear transformation algorithm [22]. *t*-SNE was used to reduce the four-dimensional data points down to a two-dimensional representation so that they can be easily visualized. The *t*-SNE 2D cluster visualization was completed using MATLAB and Statistics Toolbox Release 2019a (The MathWorks, Inc., Natick, MA, USA). *t*-SNE is a dimensionality reduction technique that is well suited to the task of visualization as it has a reduced tendency to ’crowd’ data points in the center of the lower (transformed) dimension space and can visualize clustering patterns ranging from local to global scale by maximizing the distance between dissimilar groups across the scale range. In this work, we use this approach to provide a qualitative visual assessment of the clustering solutions discovered.

**Figure 2:**
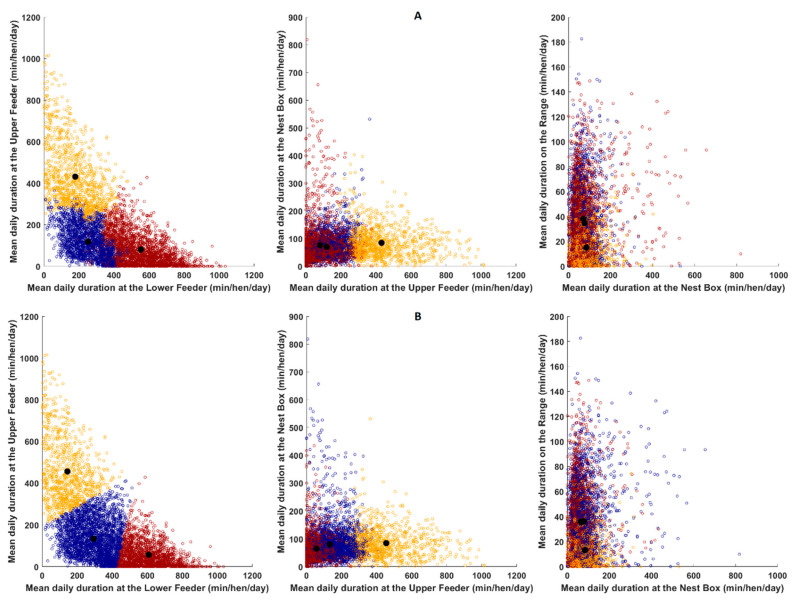
Faceted, 2-dimensional scatter plots of the clustered data across the 4 dimensions that were used to create the clustering solutions using agglomerative clustering (**A**) and K-means clustering (**B**) for the pre-laying period. Each circle represents an individual hen. The black dots represent the respective cluster centroids, plotted in the dimensions displayed for each scatter plot. Cluster membership is denoted by the marker color (cluster 1 = orange, cluster 2 = dark blue, and cluster 3 = dark red). Note the cluster distribution is different for each method.

This approach was applied to the clustering solutions for both K-means and agglomerative clustering algorithms to provide a comparison of the capability for each approach (Figure 2). In addition to the visualization using the dimensionality reduction approach, a two-dimensional scatter plot of an exemplar optimal clustering solution (K-means) was produced by plotting the data for the lower feeder, upper feeder, nest box and the range. This visualization provides an un-transformed view of the data within each cluster and demonstrates the groupings that are identified within the four discriminative dimensions.

### 2.7. Daily Feeder, Nest Box, and Range Usage 

To understand whether sub-populations remained consistent over different production periods, we further calculated the mean daily duration of different laying periods namely pre-laying period (18–22 weeks of age), peak laying period (23–33 weeks of age), late laying period (34–54 weeks of age), and end of laying period (55–74 weeks of age). To compare the daily mean duration between the groups over time, the restricted estimated maximum likelihood (REML) model was used with cluster groups, age of hens, and their interaction as the main effects and flock as a random effect.

### 2.8. Coefficient of Variation

After clustering, the hens using the mean time duration on the four different areas at 18 to 22 weeks of age, the coefficient of variation (CV) of the daily average time duration at each zone per day was used to determine the within-individual hen variability and consistency from 23 to 74 weeks of age for each hen. To show the frequency of variation, histograms of the CV of the daily average time duration at each zone were created using R software [23]. A bivariate correlation plot of the coefficient of variation for the daily duration and mean daily duration in all the hens pooled was also created using the ‘ggpubr’ package [24].

### 2.9. Bodyweight and Egg Follicle Score

To understand the differences between these clusters in the production performance, body weight was measured at 16, 22, and 74 weeks of age, while egg follicle development was assessed at the 74 weeks of age. The egg follicle was assessed using a four score system where 1 represented no active follicles, 2 represented the presence of follicles in late regression, 3 represented the presence of follicles in early regression, and 4 represented full follicle production. To distinguish the difference of sub-populations regarding their egg follicle scores, a nominal regression model was used in JMP (JMP statistical software, version 14, SAS Institute Inc., Cary, NC, USA, 1989–2020).

## 3. Results

### 3.1. K-Means and Agglomerative Cluster Characteristics

The Calinski-Harabasz criterion can be used in any number of dimensions in the dataset regardless of the distribution of the data and is therefore suitable for non-normal data as relevant for this study. Using the Calinski-Harabasz criterion and selecting the maximum index, three clusters were determined as the optimum number of clusters. The K-means algorithm selected 1470, 3473, and 2301 hens as clusters 1, 2, and 3, respectively, while the agglomerative algorithm classified 979, 3501, and 2764 as clusters 1, 2, and 3 (Table 1).

In K-means clustering method, hens of cluster 1 (*n* = 1470 hens) spent significantly more time on the upper feeding chain (498.0 ± 4.16 min/hen/day) compared to hens of cluster 2 (*n* = 3473; 143.8 ± 1.57 min/hen/day) and hens of cluster 3 (*n* = 2301; 46.28 ± 1.17 min/hen/day), respectively (*p* < 0.05). Hens of cluster 3 spent 648.5 ± 2.50 min/hen/day at the lower feeder chain compared to hens of clusters 1 and 2 (108.7 ± 2.28 and 302.4 ± 1.80 min/hen/day, respectively; *p* < 0.05). The hens from all the clusters spent least time at the range and at the nest box. Clusters 1 and 2 spent comparable time on the range (*p* > 0.05), while cluster 3 hens spent the least amount of time on range (6.18 ± 0.32 min/hen/day; *p* < 0.05). Similarly, using the agglomerative clustering method, hens of cluster 1 (*n* = 979 hens) spent significantly more time on the upper feeding chain (571.8 ± 4.60 min/hen/day) compared to hens of cluster 2 (*n* = 3501; 178.7 ± 1.94 min/hen/day) and hens of cluster 3 (*n* = 2764; 55.3 ± 1.11 min/hen/day), respectively (*p* < 0.05). Hens of cluster 3 spent 611.7 ± 2.6 min/hen/day at the lower tier feeder chain compared to hens of clusters 1 and 2 (88.9 ± 2.45 and 264.1 ± 1.86 min/hen/day, respectively; *p* < 0.05). The hens from all the clusters spent the least time at the range and at the nest box. Clusters 1 and 2 spent comparable time on the range (*p* > 0.05), while cluster 3 hens spent the least amount of time on range (4.8 ± 0.36 min/hen/day; *p* < 0.05).

### 3.2. The Agreement between the K-Means and Agglomerative Subpopulations

Of the hens classified by the K-means as clusters 1, 2, and 3 hens, the agglomerative algorithm identified 66.6%, 86.2%, and 99.2% of the hens as clusters 1, 2, and 3. Clusters were aligned between the two different approaches using the minimum distance between cluster centroids (e.g., cluster 1 from the K-means solution was aligned to the cluster from agglomerative clustering solution with the closest centroid for assessment). This deals with the random initial state used in the K-means algorithm. There was a strong agreement in the classification of hens into clusters 1, 2, and 3 between K-means and agglomerative as indicated by a kappa coefficient of 0.7794 (Table 2).

### 3.3. Visualisation of the Clusters

**Figure 3:**
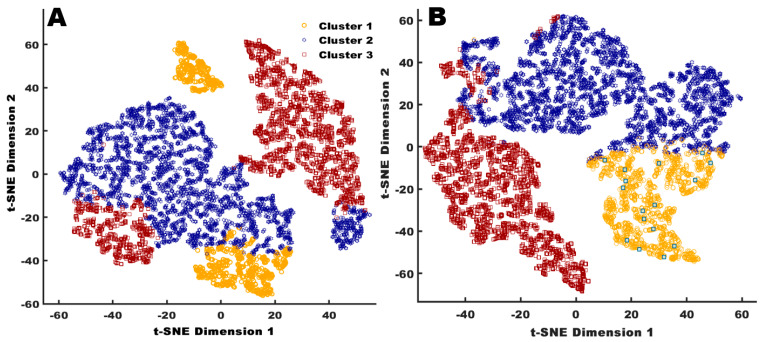
Two-dimensional scatter plot of *t*-Distributed Stochastic Neighbor Embedding (*t*-SNE) visualizing cluster assignments of individual hens by agglomerative (**A**) and K-means algorithm (**B**), where cluster 1 hens (orange circles), cluster 2 hens (dark blue star), and cluster 3 hens (dark red square), grouped according to their similarity of time duration at the feeders, nest boxes, and on the range.

To qualitatively assess the clustering solutions produced by the K-means and agglomerative approaches, the set of 2-dimensional scatter plots were produced that cover the 4 dimensions that were used by the clustering algorithms to create the groupings. As an exemplar for the visual assessment, data and groupings from the pre-laying period are presented in Figure 2. Figure 2A shows the scatter plots for the agglomerative clustering solution, and Figure 2B shows the K-means clustering solution. It can be observed that the K-means algorithm produced a solution with more discrete boundaries between clusters; however, the centroids of the clusters from both approaches largely agree, indicating that the use of the Calinski-Harabasz criterion is resulting in the selection of solutions that reflect natural groupings within that data. Figure 3 provides a set of two-dimensional visualizations that have been created using the *t*-SNE approach across the pre-laying period datasets that were used to create the clustering solutions in Figure 2. The clustering solution groupings have been overlaid (e.g., using corresponding colors with the clusters aligned using the nearest centroid) to provide an assessment of how clustering solutions follow natural groupings that are revealed by the *t*-SNE dimensionality reduction transformation. The *t*-SNE plots demonstrate that there is structure and nature grouping within the underlying data that are not evident in the multi-faceted plots. It is immediately evident that both clustering approaches produce solutions that have large contiguous blocks, with the groupings based around high-level structural elements uncovered by the *t*-SNE transform, rather than smaller scale, local, structural elements. This is important as is demonstrates that the choice of cluster selection criteria is suitable for the nature of the dataset (e.g., the scale and shape of natural groupings). The agglomerative solution in Figure 3 shows more fragmentation, with each cluster split into two components, when compared with the K-means solutions across the *t*-SNE transform. This indicates (qualitatively) that the K-means approach should provide a better reflection of the natural groupings within the data; therefore, K-means solution was used for comparing differences of the clusters in the rest of the paper.

### 3.4. Daily Feeder Usage, Nest Box and Range Access during Different Laying Periods

**Figure 4:**
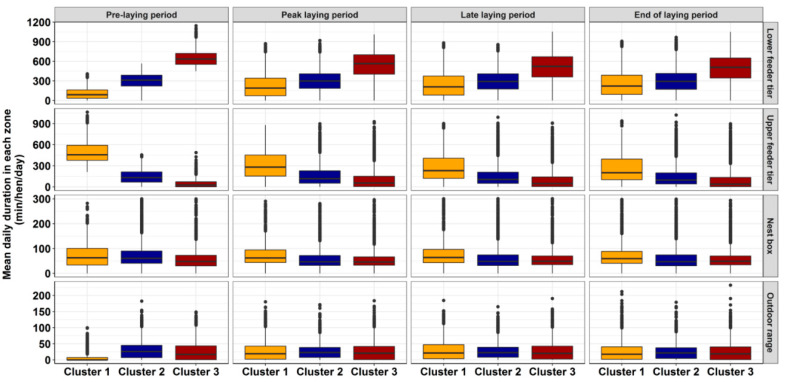
The box plots represent the time duration that hens of each cluster spent on the different tiers from during pre-laying period (18–22 weeks), peak-laying period (23–33 weeks), late laying period (34–54 weeks), and end of laying period (55–74 weeks) based on the K-means analysis.

The mean daily duration of the K-means cluster groups at different production periods are presented in Figure 4. There was a significant main effect of cluster groups, location, and age of hens, as well as a significant interaction between the cluster groups and hen age (*p* = 0.0001). During all the production periods, cluster 1 hens spent the highest time on the upper feeder tier compared to hens of clusters 2 and 3, with mean daily upper feeder duration of 311 ± 5.02 min/day/hen, 276 ± 5.01 min/day/hen, and 257 ± 4.96 min/day/hen at the peak, late, and end of laying, respectively (Figure 4). On the contrary, hens of cluster group 3 preferred to spend more time on the lower feeder with mean daily upper feeder duration of 540 ± 4.32 min/day/hen, 503 ± 4.44 min/day/hen, and 492 ± 4.51 min/day/hen at the peak, late, and end of laying, respectively, compared to clusters 1 and 2. On the contrary, there was no significant difference in the time spent on nest box tier and the outdoor range by all the cluster groups and they spent the least amount of time on the nest boxes and the range in all production periods (Figure 4).

### 3.5. Individual Variation in Daily Feeder Usage, Nest Box and Range Access

**Figure 5:**
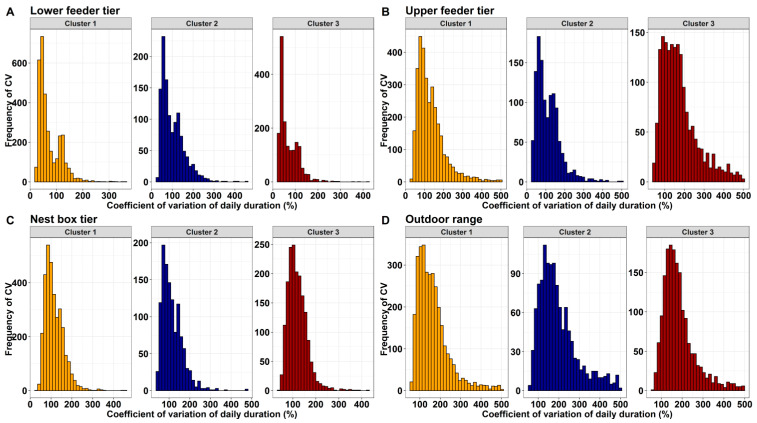
Frequency histograms of the CV for daily duration and number of visits to the range in cluster 1 (orange), 2 (dark blue), and 3 (dark red) for free-range laying hens based on the K-means cluster analysis for lower feeder zone (**A**), upper feeder zone (**B**), nest box (**C**) and outdoor range (**D**).

The distribution of the CV of the mean daily duration on the lower feeder tier, upper feeder tier, nest box tier, and outdoor range usage for the three cluster groups is shown in Figure 5. Overall, when all hens were pooled together, the highest within-individual hen variability for the daily duration as determined by the CV was observed on the upper feeder tiers and the range, a mean of 140 ± 1.02% (26–499%) and 176 ± 1.03% (55–500%) (Table 3, Figure 5), while there was a fairly lower variation of the within-individual hen variability for the daily duration observed at the lower feeder tiers and on the nest boxes with a mean of 77.5 ± 0.59% (21–452%) and 116 ± 0.57% (55–500%). Cluster 1 and 3 hens had the numerically lowest within-individual hen variability for the mean daily duration at the lower feeder of 72.2 ± 0.74% and 70.7 ± 1.03% compared to 101 ± 1.59% of cluster 2. On the other hand, cluster 3 hens had the highest within-individual hen variability on the upper feeder tier with a mean of 175 ± 2.20% compared to 132 ± 1.28% and 113 ± 1.85% of cluster 1 and 2. The hens from all the clusters had almost similar low within-individual hen variability at the nest box, while cluster 1 hens had the lowest within-individual hen variability of 164 ± 1.35% compared to 196 ± 2.64% and 185 ± 1.85% of cluster 1 and 2 hens (Table 3).

**Figure 6:**
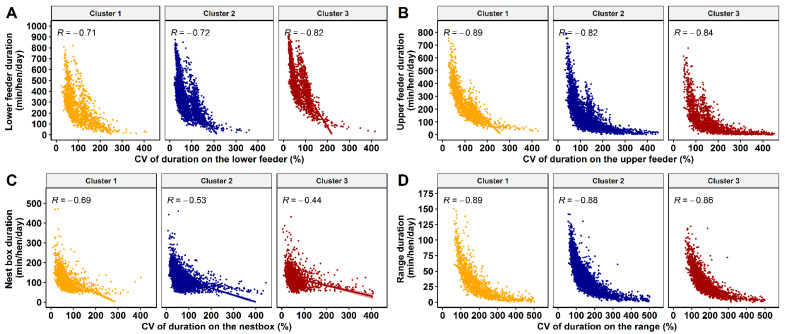
The bivariate correlation plot of coefficient of variation for the daily duration and mean daily duration for cluster 1 (orange), 2 (dark blue), and 3 (dark red) based on K-means solution for lower feeder zone (**A**), upper feeder zone (**B**), nest box (**C**) and outdoor range (**D**). Each dot represents each hen in clusters 1, 2, and 3, while the shaded area around the correlation line represents the confidence interval. All correlation coefficients are significant with *p*-values < 0.0001.

The correlation between the CV of daily mean average duration and the daily mean average duration on the different the feeders, nest boxes, and on the outdoor range of the K-means clusters is illustrated in Figure 6. Overall, the mean daily duration that hens accessed the lower feeder tier, upper feeder tier, nest box tier, and outdoor range was negatively correlated with the CV for the daily duration (Spearman’s rho = −0.66, −0.84, −0.55, and −0.88, *p* < 0.001), respectively. The CV of daily mean average duration and the daily mean average duration at the nest box had the lowest negative correlation coefficient compared to all other areas (Figure 6).

### 3.6. Bodyweight Distributions of Each Cluster

**Figure 7:**
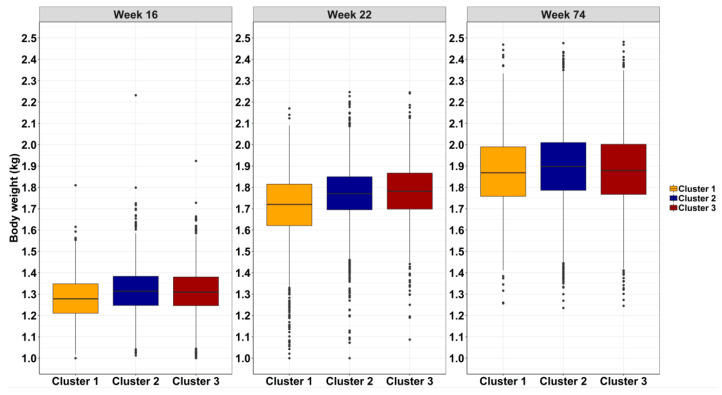
The box plots represent the body weight at 16, 22, and 74 weeks of age for cluster 1 (orange), cluster 2 (dark blue), and cluster 3 (dark red color). The lines on the box plot represent the lower quartile, median, upper quartile, and the interquartile range of body weight of each cluster based on the K-means solution.

The difference in body weight for the three clusters is presented in Figure 7 at 16, 22, and 74 weeks of age. For bodyweight, there was an overall observed significant effect of the cluster group and age of the hens on mean body weight (*p* = 0.0001). At 16 weeks of age, cluster 1 had significantly lower body weight (1.28 ± 0.003 kg) compared to clusters 2 and 3, with mean body weights of 1.31 ± 0.002 kg and 1.31 ± 0.003 kg, respectively. This pattern was also observed at 22 weeks of age, where cluster 1 hens had a significant lower body weight of 1.63 ± 0.010 kg compared to clusters 2 and 3 with mean body weights of 1.71 ± 0.006 kg and 1.71 ± 0.008 kg, respectively. (*p* = 0.0001; Figure 7). On the contrary, there was no significant difference of body weight between the hen clusters at 74 weeks of age (*p* > 0.05; Figure 7).

### 3.7. Egg Follicles

The results for egg follicles conditions are presented in Table 4. There was a flock effect on the egg follicle score (*p* = 0.0092, Table 4). There was no main effect of the flock sub-population on their egg follicle scores (*p* = 0.4133) nor any interaction detected. 

## 4. Discussion

The automated classification of animals, such as laying hens, is a novel method that can support farmers in decision-making to optimize their egg production, taking the behavior and needs of the different flock sub-populations (clusters) into account, rather than relying on the conventional information based on observing the performance of the flock average. In this experiment, we used visualization based upon the t-SNE dimensionality reduction technique to reveal the natural groupings within the flock data and demonstrated the ability of the clustering algorithms to automatically assign hen data points according to these natural groupings. The results demonstrated that the behavior between individual hens, as well as between sub-population, can be significantly different when accessing the outdoor range but also key resources, such as the nest boxes and feeder chains. The formation of the distinct clusters was associated with the location of hens in different areas of the aviary system, which would allow for different management strategies based on hen location to manage an even use of resources. For example, in order to optimize range and aviary usage, it is important to identify hen movement patterns and space usage of individual inside the shed to avoid competition and smothering. For hens that spent most of their time on the upper tier, access to ramps may be provided to encourage the use of the lower tier and the range. In addition, allowing for additional resources on the upper feeder tier, such as drinker lines or even nest boxes, may significantly improve hen welfare and performance and further research in this area might significantly change the current design of aviaries for the benefit of the hens. We previously described statistically significant differences in egg production, health, and welfare of various range use flock sub-populations, which allowed for determining hens that are economic more beneficial compared to under-performing groups [25,26]. Access to resources alone can be a significant contributor to inadequate performance, whereas the genetic potential of the animal cannot be utilized. One major limitation of this study was that, due to the commercial nature of the study and the use of feeder chains that ran throughout the shed, we were not able to quantify the amount of feed intake, including the impact of daily feed intake on body weight/egg performance. This is relevant as malnourished hens, non-productive hens, or egg misplacement can be a sign of compromised health but will also affect the economic return of the egg producer. While it has been shown previously that range use could be associated with increased egg performance at the beginning but not the end of lay, clusters 2 and 3 (hens that initially spent the longest duration on the range) had the lowest percent of hens in full lay at 74 weeks of age, but this was not statistically significant (Table 4) [26]. Rationale for this may include that range usage did not differ significantly between the clusters at the end of the laying period and, as such, impact of UV light exposure on ovulation rate might be comparable [27]. 

Alternatively, the high variation of movement patterns observed may have reduced the impact that extreme range use/shed use may have had on performance parameters, leading to non-significant findings in the present study. However, we previously detected a positive correlation between range use and lower feeder chain use, which can be confirmed using the K-mean clustering, whereas hens of cluster 3 that spent the most time on the range were also the predominant users of the lower feeder chain [6]. Interestingly, the investigation of movement patterns provides new insight into the flock dynamics and, to our best knowledge, have not been reported to date: Hens that were more consistent with their average time spent at each location stayed for longer duration at each location compared to those hens that had inconsistent movement patterns. The identification of ‘routine’ behavior patterns can be essential for flock management, using temporal change detection algorithms to generate alerts when hen dynamics change may allow management to intervene to prevent uncoordinated mass movements that lead to smothering [12]. In addition, dysregulated individual hen movement and time budgets increase the likelihood of hen injury due to competition for resources, such as perching space or feed, leading to severe welfare implications, such as keel bone and toe fractures, inter-hen aggression, and, subsequently, hen death [28,29,30,31,32,33,34]. The data represented in this study demonstrates that clustering the hens according to their time budgets had no impact on individual movement variation, indicating the large variety of how hens express their choice of movement. For example, the CV for the duration that hens accessed the range varied from 55.4 to 500% with a mean of 176 ± 1.03% (Table 3). Similar findings have been observed by Larsen et al., 2017 [4], where the variation was 18 to 361% for layers exposed to the range for 12 consecutive days at 21 and 41 weeks of age. Regardless of the cluster group, some hens showed variation in their average time spent on the different zones, which might suggest that hens have spatial preferences, while some individual hens were more adventurous and rarely followed the same movement pattern [35,36]. The formation of hen clusters with consistent time budgets may indicate that some flock sub-populations prefer certain resources, while being less orientated toward territorial behaviors. Individual hen preferences for the different resources may be explained by the individual difference in their physiology, phenotypic appearance, epigenetics, early-life experience, or learning abilities [37].

Understanding the individual’s motivation to access or avoid certain resources will be paramount to train hens to use the available equipment and space to optimize welfare and production outcomes [38]. Being able to trace and monitor hen behavior and recognize patterns, as well as investigating causalities, allows the identification of vulnerable individuals and sub-population in the future. These may include hens subject to keel bone damage, severe feather pecking, cannibalism, or smothering [39,40]. Furthermore, offering different diets or feed additives through different feeder lines may directly target the different requirements for hens that favor these specific locations.

In the ever-increasing farm size and increased consumer awareness, producers are often challenged with having to project how flock management decisions will affect production performance and welfare outcomes in a complex system. This work has demonstrated that hen movement data has the potential to be used for prediction of health and welfare outcomes, especially when hens are showing consistency in their movement patterns. With the development of predictive models, we should also recognize the complexity inherent in behavior, physiological, and biological systems. In order not to be blindsided by unforeseen outcomes, a methodical, all-inclusive technique of analyzing, modeling, and simulating complex behavioral data to predict anticipated outbreaks is warranted. However, the availability of comprehensive models of behavior, physiological, and biological systems combined with RFID data are currently limited [41].

Feeding and nesting are highly motivated behaviors, thus being able to determine the maximal percentage of hens that are accessing the resources at any given time can allow for adequate changes in flock management, such as feeding time manipulations or adjusting the nest box opening and closing times. It is not only of the highest degree of interest to investigate how technology can be used to support underperforming sub-populations but also how to ensure that over-performing hens are truly nurtured to their best long-term care. Consequently, it is crucial to develop and test approaches to continuously monitor hen movement and activity, which can then turn into solutions that allow for real-time decision-making and alert systems if unusual patterns and overcrowding are observed. 

## 5. Conclusions

Three flock sub-populations were identified in the aviary system, and these sub-populations expressed an uneven load on the resources (e.g., feed chains, nest boxes, range use). Unsupervised and machine learning algorithms and data visualization (based on dimensionality reduction) identified hen groupings related to time budgets. While we demonstrated a technique that could be the basis for autonomous monitoring systems, further understanding of the impact of the variable use of the resource within and between individuals can improve management practices, shed design, and hen welfare, allowing for a vital opportunity for forthcoming investigation. Additional analysis of the data using classification models based on support vector machines, artificial neural networks and decision trees is warranted to detect the contributions of other relevant parameters for hen performance.

## Figures and Tables

**Table 1:** The descriptive statistics of the K-means and agglomerative clusters of the free-range flocks using daily mean time duration in each zone from 18–22 weeks of age (pre-laying period). CV = coefficient of variation.

Summary Statistics		Average Lower Feeder Time (Min/Hen/Day)		Average Upper Feeder Time (Min/Hen/Day)		Average Nest Box Time (Min/Hen/Day)		Average Range Use Time (Min/Hen/Day)	
		K-Means	Agglomerative	K-Means	Agglomerative	K-Means	Agglomerative	K-Means	Agglomerative
Cluster 1	Mean ± SEM	108.7 ± 2.28	88.9 ± 2.45	498.0 ± 4.16	571.8 ± 4.60	71.9 ± 1.37	68.2 ± 1.58	6.18 ± 0.32	4.80 ± 0.36
	SD	87.4	76.7	159.7	144.1	52.5	49.3	12.1	11.4
	Skewness	0.85	0.99	0.89	0.87	1.41	0.79	3.07	3.88
	CV	80.3	0.48	32.1	0.14	73.0	0.44	196.2	19.4
	Median	89.7	65.3	458.3	539.6	63.1	60.5	0.01	0.00
	N	1470	979	1470	979	1470	979	1470	979
Cluster 2	Mean ± SEM	302.4 ± 1.80	264.1 ± 1.88	143.8 ± 1.57	178.7 ± 1.94	78.1 ± 1.16	72.88 ± 0.89	30.0 ± 0.45	26.59 ± 0.43
	SD	106.2	109.9	92.7	114.6	68.4	52.6	26.6	25.4
	Skewness	−0.31	−0.17	0.42	0.32	3.54	2.64	1.02	1.10
	Kurtosis	−0.66	−0.436	−0.55	−0.865	18.70	12.2	1.19	1.39
	Median	312.1	273.5	132.2	164.9	61.2	61.1	26.1	22.4
	N	3473	3501	3473	3501	3473	3501	3473	3501
Cluster 3	Mean ± SEM	648.5 ± 2.50	611.7 ± 2.61	46.3 ± 1.17	55.3 ± 1.11	59.0 ± 1.05	69.1 ± 1.38	26.8 ± 1.20	27.9 ± 0.57
	SD	119.8	137.2	56.2	58.5	50.2	72.4	29.6	30.1
	Skewness	0.70	0.47	1.80	1.03	2.61	3.57	1.20	1.17
	Kurtosis	0.32	0.001	4.89	0.178	11.9	18.5	0.94	0.866
	Median	635.1	602.1	26.1	37.0	48.0	51.4	16.9	18.8
	N	2301	2764	2301	2764	2301	2764	2301	2764

SD represents standard deviation; SEM represents standard error of the mean.

**Table 2:** Contingency table of the hen classification by the agglomerative and K-means clustering algorithms with Kappa agreement statistics.

Clustering Algorigthm		Agglomerative Clustering			Total N
		Cluster 1 N (%)	Cluster 2 N (%)	Cluster 3 N (%)	
K-means	Cluster 1	979 (66.6)	491 (33.4)	0 (0)	1470
	Cluster 2	0 (0)	2992 (86.2)	481 (13.9)	3473
	Cluster 3	0 (0)	18 (0.78)	2283 (99.2)	2301
	Total	979	3501	2764	7244
Kappa coefficient	Kappa	SEM	Lower 95%	Upper 95%	
	0.7794	0.0065	0.7667	0.7922	

**Table 3:** The descriptive statistics of the CV for the daily duration of the three clusters detected in commercial free-range flocks.

Cluster	Summary Statistics	CV of Mean Lower Feeder Duration (%)	CV of Mean Upper Feeder Duration (%)	CV of Mean Nest Box Duration (%)	CV of Mean Range Use Duration (%)
Cluster 1 (*n* = 979)	Mean ± SEM	72.2 ± 0.74	132 ± 1.28	115 ± 0.77	164 ± 1.35
	SD	42.2	73.4	44.2	77.2
	Maximum	360	499	461	500
	Minimum	21.0	31.5	32.4	55.3
	Median	55.4	113	105	147
Cluster 2 (*n* = 3501)	Mean ± SEM	101 ± 1.59	113 ± 1.85	114 ± 1.44	196 ± 2.64
	SD	55.3	64.6	50.2	92
	Maximum	452	496	471	500
	Minimum	23.8	26.1	38.5	59.6
	Median	84.9	98.7	103	175
Cluster 3 (*n* = 2764)	Mean ± SEM	70.7 ± 1.03	175 ± 2.20	120 ± 1.02	185 ± 1.85
	SD	43.0	91.9	42.8	77.4
	Maximum	416	497	432	500
	Minimum	21.0	44.3	43.9	65.0
	Median	53.6	155	113	167
Pooled (*n* = 7244)	Mean ± SEM	77.5 ± 0.59	140 ± 1.02	116 ± 0.57	176 ± 1.03
	SD	46.8	80.8	45.11	81.4
	Maximum	452	499	471	500
	Minimum	21.0	26.1	32.4	55.4
	Median	60.7	123	107	158

**Table 4:** The proportion of hens with different egg follicle scores of clusters 1, 2, and 3 in free-range laying hens at 74 weeks of age based on the K-means analysis.

Sub-Population	Egg Follicle Observation; N (%)			
	No Follicles	Late Regression	Early Regression	Full Egg Production
Cluster 1	55 **(1.58)**	43 **(1.24)**	107 **(3.08)**	3266 **(94.1)**
Cluster 2	35 **(2.38)**	19 **(1.29)**	50 **(3.40)**	1366 **(92.9)**
Cluster 3	44 **(1.92)**	34 **(1.48)**	75 **(3.27)**	2144 **(93.3)**
Pooled	134 **(1.85)**	96 **(1.33)**	232 **(3.21)**	6776 **(93.6)**
*p*-value Cluster		0.4133		
*p*-value Flock		0.0092		
*p*-value Flock × Cluster		0.7167		

The numbers with bold face represent the percentage proportion of the hens while the numbers without bold face represent the actual count of hens.
